# Using health worker opinions to assess changes in structural components of quality in a Cluster Randomized Trial

**DOI:** 10.1186/1472-6963-14-282

**Published:** 2014-06-28

**Authors:** Naomi Muinga, Philip Ayieko, Charles Opondo, Stephen Ntoburi, Jim Todd, Elizabeth Allen, Mike English

**Affiliations:** 1 KEMRI/Wellcome Trust Research Programme, Nairobi, Kenya; 2 Department of Population Health, London School of Hygiene and Tropical Medicine, London, UK; 3 Department of Medical Statistics, London School of Hygiene and Tropical Medicine, London, UK; 4 Department of Paediatrics, University of Oxford, Oxford, UK

**Keywords:** Quality improvement, Child health, Paediatrics, Health services research

## Abstract

**Background:**

The ‘resource readiness’ of health facilities to provide effective services is captured in the structure component of the classical Donabedian paradigm often used for assessment of the quality of care in the health sector. Periodic inventories are commonly used to confirm the presence (or absence) of equipment or drugs by physical observation or by asking those in charge to indicate whether an item is present or not. It is then assumed that this point observation is representative of the everyday status. However the availability of an item (consumables) may vary. Arguably therefore a more useful assessment for resources would be one that captures this fluctuation in time. Here we report an approach that may circumvent these difficulties.

**Methods:**

We used self-administered questionnaires (SAQ) to seek health worker views of availability of key resources supporting paediatric care linked to a cluster randomized trial of a multifaceted intervention aimed at improving this care conducted in eight rural Kenyan district hospitals. Four hospitals received a full intervention and four a partial intervention. Data were collected pre-intervention and after 6 and 18 months from health workers in three clinical areas asked to score item availability using an 11-point scale. Mean scores for items common to all 3 areas and mean scores for items allocated to domains identified using exploratory factor analysis (EFA) were used to describe availability and explore changes over time.

**Results:**

SAQ were collected from 1,156 health workers. EFA identified 11 item domains across the three departments. Mean availability scores for these domains were often <5/10 at baseline reflecting lack of basic resources such as oxygen, nutrition and second line drugs. An improvement in mean scores occurred in 8 out of 11 domains in both control and intervention groups. A calculation of difference in difference of means for intervention vs. control suggested an intervention effect resulting in greater changes in 5 out of 11 domains.

**Conclusion:**

Using SAQ data to assess resource availability experienced by health workers provides an alternative to direct observations that provide point prevalence estimates. Further the approach was able to demonstrate poor access to resources, change over time and variability across place.

## Background

Common childhood illnesses including pneumonia, malaria, and diarrhea together with illness in the newborn period remain major contributors to child mortality in low-income countries [1]. Hospital care of severe illnesses may help improve survival, and clinical guidelines to help direct delivery of the most appropriate interventions have been provided by the World Health Organization (WHO) [2,3] and nationally in Kenya [4]. However, the ability to provide effective care is often undermined by lack of appropriate resources or poor organisation of care [5-7]. In addition, the simple act of being admitted may carry risks from nosocomial infection linked to inadequate resources for and implementation of infection prevention efforts [8].

The ‘resource readiness’ of health facilities to provide effective services is captured in the structure component of the classical Donabedian paradigm often used for assessment of the quality of care in the health sector [9]. The other components of the paradigm are process of care and their outcomes. According to this paradigm, structural aspects of quality constitute: the physical infrastructure, human resources, the availability of diagnostic tools or services, drugs and other consumables as well as the organisational arrangements made to provide care. It is assumed that such inputs, if available and appropriately employed in processes of care- through the actions of health workers- may lead to desired outcomes [9]. This report focuses on structural aspects of quality of care, and reports on services provided to children and newborns in Kenyan hospitals [10]. In particular, we assess elements of structure such as hygiene products or drugs whose availability may change over relatively short periods of time (days or weeks) [11].

The most common approach to assessing elements of structure is by periodic inventories [12]. This way the presence (or absence) of equipment or drugs is confirmed by physical observation or by asking those in charge to indicate whether an item is present or not. The Service Provision Assessments (usually part of Demographic and Health Surveys) are examples of large scale inventories that cover aspects of structure across many spheres of low-income country health systems [13]. Some inventories cover specific areas of services or even single entities such as the availability of appropriate antimalarial drugs and so on [14]. By their nature, these surveys only establish what is present at the time of the assessment – a point assessment (or prevalence if data are aggregated across places). It is then assumed that this point observation is representative of the everyday status. However the availability of an item (particularly those that are consumables) may vary. Items may be available immediately after procurement, but may not be available shortly after if the supply was inadequate. Arguably therefore a more useful assessment for resources would be one that captures this fluctuation in time. Here we report an approach that may circumvent these difficulties.

## Methods

The data used in this report were collected as part of a cluster randomized trial of a multifaceted intervention aimed at improving paediatric inpatient care [15]. The details of the trial are presented elsewhere. Briefly, eight rural hospitals (H1 to H8) were chosen purposefully from four of Kenya’s eight provinces to provide some representation of the variety of typical, rural hospital settings encountered in Kenya [16]. Hospitals admitting a minimum of 1,000 children and conducting at least 1,200 deliveries per year were eligible for inclusion. After obtaining the hospitals’ assent four hospitals were allocated to a full (intervention group, hospitals H1–H4) and four to a partial (control group, hospitals H5–H8) package of interventions using restricted randomization. This study was conducted with the approval of the Kenya Medical Research Institute (KEMRI) Ethics Review Committee (reference number SSC 991) in Kenya.

The interventions applied and their implementations are described in full elsewhere [15,17]. In summary, hospitals received (in both groups unless specified): (1) regular hospital assessment through surveys conducted six monthly, followed by (2) written feedback (all sites) with face-to-face feedback in intervention sites only; (3) training, of 5.5-days duration for 30–40 health workers of all cadres in intervention sites and 1.5 days in control sites approximately 6–10 weeks after baseline surveys [4]; (4) provision of clinical practice guidelines introduced with training; (5) job aides. Intervention sites received in addition: (A) an external supervisory process, and (B) identification of a full-time local facilitator (a nurse or diploma-level clinician) responsible for promoting guideline use and on-site problem solving [10]. Supervision visits were approximately two to three monthly, but facilitation remained in place throughout the 18 months.

This design thus compared two alternative intensities of intervention, although we refer to one arm as the “control”. It is worth noting that no financial or commodity resources were supplied. Thus changes that occurred might have reflected improvements across the health sector or potentially indirect effects of intervention. For example the intervention may have resulted in a greater demand for certain drugs, and may have influenced the hospitals management response to these demands. That this happened is supported by previous qualitative research [18] and by analysis of resource inventory data [15]. Here we explore whether resource availability assessed from health worker reports was also useful in exploring this effect.

### Health worker reported availability

#### Scope of inquiry

The purpose of the assessment was to establish the availability of items necessary for provision of effective care to sick newborns or children based on health workers’ experience. These items were identified from The WHO hospital assessment tools and national guidelines on provision of care for children in hospitals. The items necessary for provision of care varied by the area where the care is given. In Kenya care is provided to children and newborns in various areas within the hospital – in the maternal and child health clinic (MCH) where routine and walk-in (acute) services are provided in the outpatient area, the inpatient paediatric ward (PW) and the maternity ward with newborn nursery (NN). On the basis of key guidelines and the organisation of previous tools ([10]) we therefore defined, *a priori*, specific items for investigation relevant to each clinical area (PW = 34, NN = 20; MCH = 12) [19]. Where items were common across clinical areas (for example availability of soap for hand washing) the same question was used. Our *a priori* reasoning was that items represented a total of 14 pre specified logical groupings across the three areas. These 14 logical groupings encompassed items related to infection prevention such as hand-washing, ward cleanliness, and patient isolation; the availability of therapeutic interventions: oxygen; recommended first line drugs; recommended second-line drugs; therapeutic or supportive feeding; and emergency fluids or blood. There seem no standard approaches to assessment of resource availability in any of these logical groupings.

#### Structure of questionnaires

Inventories such as one reported in [15], consider an item present or not; those that report on stockouts often employ questionnaires, where the availability of an item is scored on semi-quantitative scales [20], often a likert scale. It is the latter that we use in this report. In accordance with standards in developing questionnaires we aimed for a simple, concise but comprehensive and unambiguous questionnaire. For each item the health worker was asked to consider the ten most recent occasions that they needed to use an item and for how many of these was the item available. They reported this on a 11 point (0–10) likert scale. An option for ‘don’t know’ was also provided for health workers who had no relevant experience on which to base a response, for example a worker may not have had sufficient experience related to availability of blood for transfusion. The questionnaire was divided in to 3 sections, representing the 3 clinical areas where children are cared for. A health worker was required to respond only to clinical areas that s/he was familiar with or working in. The questionnaire was designed for self administration and instructions were written as a preamble. The questionnaire was pilot tested on 50 health workers from a hospital not involved in the study to check relevance and comprehension and amendments made to promote clarity as required in line with good practice [21].

#### Procedure

Paediatric and neonatal care is typically provided in each of the three clinical areas by between 1 to 4 clinicians (mostly junior doctors and non-physician clinicians) and 6 – 15 nurses (detailed descriptions of hospitals studied can be found elsewhere [10]). Nurses attached to PW and NN work in shifts. The opportunity to collect data was limited to hospital surveys, encompassing all data collection related to the trial, that were conducted over periods of 2 weeks in each site. During this period health workers on duty at the three clinical sites were invited to complete an SAQ relevant to their clinical areas, random selection from a staff list was not deemed feasible. By accepting to answer a questionnaire, consent was assumed to have been given by the health worker. One survey team member distributed the SAQs and followed up staff during the survey period to collect them. To ensure that the health workers felt that their identity was protected we collected data on cadre only and not age, sex or other demographic details. The aim was to collect 6 SAQs per clinical area representing a mixture of clinicians and nurses and thus a total of 18 per hospital and 144 per survey round. As this was exploratory work for which we had no prior data to inform sample size estimates such sampling was based on what was considered feasible and with a view to *post-hoc* exploratory factor analysis for which at least 100 observations are considered adequate [22]. Surveys were undertaken at baseline (pre-intervention) and then 6 and 18 months later using the same questionnaires.

#### Data handling and analysis

Data collected on SAQ were double entered and verified using Microsoft Access 2003®. Data cleaning was done by excluding ‘Don’t know’ responses from the analysis. Analyses were done using Stata, version 11 (Stata Corp.).

There were 5 items that were common across the three clinical areas (MCH, PW, and NN). For these the item responses from all respondents from all the three areas were pooled within hospital. The mean score for each item for each hospital was calculated for the three different periods: baseline, 6 and 18 months (after checking that the assumption of normality was not violated). We considered weighting the mean score -for these items that were common to different clinical areas- on the number of responses from the different clinical areas. This made little difference to the unweighted results and the latter are therefore used throughout. Mean scores were similarly calculated for specific items. The mean scores are interpreted as the mean number, out of ten occasions, that an item was available when the health worker needed it.

Exploratory factor analysis (EFA), a data reduction technique, was used to explore alternative item groupings as a precursor to developing summary structure scores from item groupings [23]. Data from all the surveys were pooled for the exploratory factor analysis as it was assumed that the underlying relationships between items represented by latent factors should not be influenced by time. EFA was however carried out using data collected from each clinical area independently as items differed. We used the Iterated Principal Factors estimation method in Stata 11 and oblique rotation for extraction of factors. Oblique rotation was selected as it was assumed that the factors to be generated were correlated [22].

**Figure 1:**
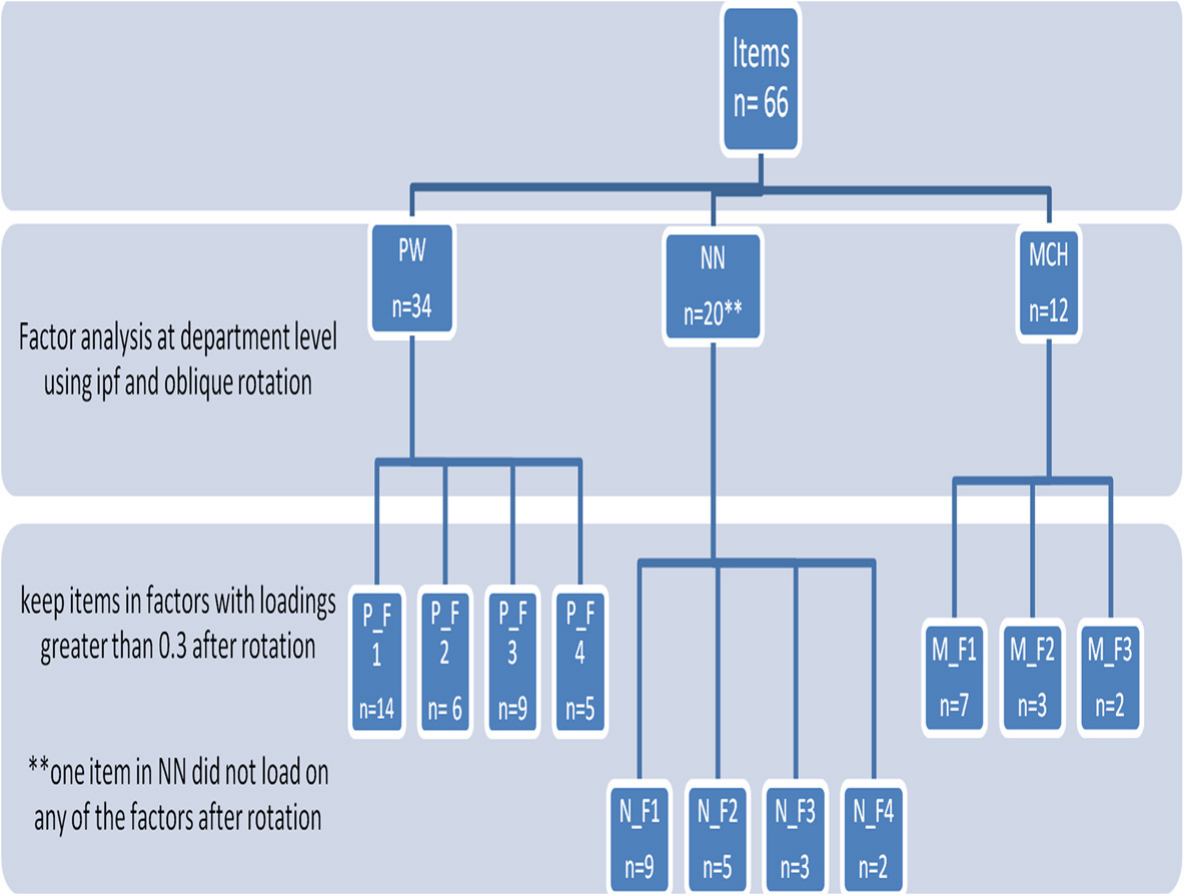
**Number of items per factor/domain extracted from EFA.** The figure illustrates factor extraction from the exploratory factor analysis carried out at the department level. The number of items in each of the factors in each of the departments is shown on the lower level of the chart.

The results of the exploratory factor analysis were used to identify *post hoc* ‘domains’ based on the latent factors discerned from the data. Items were then assigned to the domain for which they had the highest factor loading. Figure 1 shows the number of items per factor/domain in each of the clinical areas. Domain names were given that best reflected the set of items linked to a domain and our understanding of their co-location. A detailed table of all the items in each of the domains is provided in Additional file 1.

All items assigned to a domain were then used to calculate a domain specific, survey specific, summary score by summing all raw scores for domain linked items across all respondents in a department within a hospital. This provided an average score (0–10 range) for each domain in each hospital area for each hospital and each survey. Items that had a loading of less than 0.3 for any domain were not assigned to a domain and not used in the calculation of summary domain scores [22]. These domain/area/hospital average scores were further averaged at hospital group level with 95% CI based on n = 4 observations. Contrasting intervention and control group mean scores allows an exploration of change across surveys between groups and use of difference in difference calculations to explore intervention vs. control group mean changes.

## Results

The results from the parent cluster RCT have been published elsewhere [15,24]. SAQ were collected from a total of 1,156 health workers: 390 at baseline (range per hospital 47 to 50); 389 at 6 months (range 45–50) and 377 at 18 months (range 27–51). The majority of respondents were nurses (688/1,156, 59.5%) and SAQ were returned from health workers in MCH(46%), more often than from PW(24%) or NN(24%). Respondent characteristics are further described in Tables 1 and 2. On average, respondents in MCH (31.6 months) had worked longer than those responding from PW (15.9 months) or NN (14.9 months).

**Table 1:** Distribution of health workers surveyed and the average number of months worked

**Health worker type**	**PW**	**NN**	**MCH**	** Total**
	**N**	**N**	**N**	** N (%)**
**Months worked**	**Mean months worked (sd)**	**Mean months worked (sd)**	**Mean months worked (sd)**	
**Nurses**	165	212	294	688(59.52)
	17.58(16.41)	17.95(16.55)	25.66(32.1)	
**Clinical officers**	70	33	179	273(23.62)
	19.06(50.37)	6.88(16.02)	42.15(59.68)	
**Medical officers**	44	51	8	85(7.35)
	6.25(11.4)	5.37(9.56)	18.5(22.51)	
Other cadres*	37	25	41	110(9.52)
	14.54(21.7)	19.44(26.24)	31.05(47.58)	
**Total**	316	321	522	
	15.97(28)	14.93(17.35)	31.63(45.14)	

*Other cadres include: Paediatricians, lab technologists, nutritionists, oral health, orthopaedic, pharmacists, pharmacy technologists, physiotherapists and subordinate staff.

**Table 2:** Responses per survey and clinical area

		**Intervention Hospitals**				**Control Hospitals**			
**Survey**	Total**	**Median (range)**	**Range per clinical area**			**Median (range)**	**Range per clinical area**		
			**Median (range)**				**Median (range)**		
			**PW**	**NN**	**MCH**		**PW**	**NN**	**MCH**
0 months	390 (33.74%)	47.5 (36–50)	11 (8–13)	11.5 (11–13)	25.5 (12–28)	47.5 (45–50)	11 (8–18)	11 (5–13)	26 (23–27)
	Total	181	43	47	91	190	48	40	102
6 months	389 (33.65%)	48 (40–48)	12 (8–12)	12 (9–16)	23.5 (17–27)	47.5 (44–50)	13.5 (11–15)	13.5 (7–17)	21 (18–25)
	Total	184	44	49	91	189	53	52	85
18 months	377 (32.61%)	45.5 (42–50)	11.5 (5–13)	13 (9–17)	21.5 (17–30)	48 (25–51)	13.5 (6–16)	10.5 (7–15)	22 (12–24)
	Total	183	41	52	90	172	49	43	80
Total	1,156	588				568			

**Some health workers reported to provide services in 2 areas (n = 49) and some in all 3 areas (n = 8).

Mean scores (0 to 10 absolute scale) for the five items that could be assessed for each department and thus pooled across all respondents in a hospital are presented in Table 3 with their confidence intervals for surveys at baseline (0 months), 6 and 18 months post-intervention for control and intervention hospitals. These data (absolute scores) show considerable variation of the items at baseline with ability to provide oxygen at the right flow rate and availability of oxygen having the lowest scores (<5/10) for both intervention and control hospitals. Items to do with cleanliness scored better in control hospitals than intervention hospitals at baseline. The availability of water from taps changed over time with the control hospitals continuing to have a higher score at subsequent surveys than intervention hospitals. Ability to provide accurate oxygen flow rates remained at a score of less than 5 (in <50% occasions was this possible) at the second survey (6 months) for both intervention and control hospitals with a slight improvement recorded at 18 months. The availability of bolus glucose for both intervention and control hospitals consistently scored above 8/10 for all hospitals after the onset of intervention. However for none of these essential items did scores reach 10. Scores for common items for each hospital and survey (provided with confidence intervals in Additional file 2) show considerable variability at baseline across hospitals, and commonly show improving scores where these were low at baseline but with considerable heterogeneity in improvement.

**Table 3:** Means for common items at survey 1, 2 and 4, taken from the SAQ using a scale of 0–10 to show availability

		**Survey**		
**Item**	**Group**	**Baseline**	**6 months**	**18 months**
Water from taps to wash your hands between patients	Intervention	4(3.53-4.48)	5.57(5.06-6.09)	6.13(5.66-6.6)
	Control	6.73(6.3-7.16)	7.27(6.86-7.68)	7.1(6.67-7.54)
Soap/disinfectant to clean your hands between patients	Intervention	4.94(4.41-5.47)	5.36(4.8-5.91)	6.69(6.21-7.17)
	Control	7.09(6.62-7.57)	7.63(7.17-8.09)	6.9(6.41-7.39)
Availability of oxygen when needed	Intervention	4.64(4.07-5.2)	6.72(6.13-7.32)	7.59(7.1-8.08)
	Control	4.18(3.55-4.81)	5.77(5.12-6.42)	5.43(4.81-6.04)
Ability to provide oxygen at a flow of 2 l/min(for PW and MCH) or 1 l/min(NN) to each individual patient	Intervention	3.14(2.6-3.67)	4.98(4.34-5.61)	6.46(5.91-7.02)
	Control	1.86(1.38-2.35)	3.45(4.76-166)	4.07(3.45-4.69)
Bolus glucose – number of times drug is immediately available to treat hypoglycaemia (within 2 minutes)	Intervention	5.87(5.37-6.36)	8.13(7.7-8.56)	8.56(8.18-8.95)
	Control	7.32(6.78-7.86)	8.58(8.2-8.95)	8.4(7.96-8.83)

Exploratory factor analysis conducted on responses pooled within clinical areas (PW, NN, and MCH) and across surveys provided some support for our *a priori* logical groupings but items were grouped after factor analysis into less *post-hoc* domains. The clinical areas specific EFA suggested the following domains:

1) PW - 4 domains (underlying factors, in order from 1 to 4) that could be considered to represent: essential drugs/emergency resources, nutrition and supportive care, cleanliness and second-line drugs.

2) NN 4 domains representing: supportive care; common drugs; resuscitation and warming; and cleanliness.

3) In MCH 3 domains representing: hygiene and emergency care; drugs; and oxygen.

The summary scores for each domain for each hospital group at each time point, with their respective confidence intervals, are displayed in Table 4. These data suggest an improvement in 8/11 domains that were generated in the factor analysis. Improvements in both intervention and control groups were recorded in: nutrition and supportive care and second-line drugs (PW); supportive care, common drugs and resuscitation and thermal equipment (NN); hygiene and emergency care, drugs and oxygen availability (MCH). In three domains: essential drugs/emergency resources (PW) and cleanliness (PW and NN) an improvement was found in the intervention hospitals only.

**Table 4:** Domain means with confidence intervals, mean difference (s4 – s1) for each group (intervention and control) and mean difference of difference for control and intervention hospitals

**Domains**		**Survey mean (ci)**				
		**Baseline**	**6 months**	**18 months**	**Mean S**_**b**_**-S**_**18mth **_**(ci)**	**mean M**_**i**_**-M**_**c **_**(ci)**
**PW**						
Essential drugs/emergency resources	Intervention	7.15(6.59-7.72)	7.94(7.4-8.48)	8.56(8.07-9.04)	1.4(0.66-2.15)	0.99 (−0.14-2.12)
	Control	7.63(6.95-8.3)	7.99(7.49-8.49)	8.04(7.46-8.62)	0.41(−0.46-1.28)	
Nutrition and supportive care	Intervention	2.8(2.11-3.49)	6.78(6.06-7.51)	6.84(6.11-7.57)	4.04(3.05-5.03)	2.21 (0.81-3.62)
	Control	3.12(2.37-3.88)	6.17(5.31-7.02)	4.95(4.26-5.65)	1.83(0.82-2.84)	
PW cleanliness	Intervention	4.89(4.41-5.38)	5.19(4.65-5.73)	6.99(6.48-7.49)	2.09(1.4-2.78)	2.16 (1.07-3.25)
	Control	5.36(4.76-5.96)	5.95(5.38-6.52)	5.29(4.63-5.95)	−0.07(−0.94-0.81)	
Second-line drugs	Intervention	3.18(2.52-3.83)	5.93(5.34-6.53)	7.64(7.05-8.22)	4.46(3.59-5.33)	0.88 (−0.34-2.11)
	Control	2.19(1.59-2.79)	4.33(3.69-4.97)	5.77(5.13-6.41)	3.58(2.7-4.46)	
**NN**						
Supportive care	Intervention	4.25(3.71-4.8)	6.37(5.76-6.98)	6.64(6.03-7.26)	2.39(1.57-3.2)	−0.08 (−1.25-1.09)
	Control	3.52(2.93-4.12)	5.67(5.12-6.21)	5.99(5.43-6.55)	2.47(1.67-3.26)	
Common drugs	Intervention	4.9(4.33-5.47)	6.37(5.76-6.98)	6.96(6.27-7.66)	2.07(1.17-2.96)	−0.01 (−1.32-1.3)
	Control	5.34(4.73-5.95)	5.67(5.12-6.21)	7.42(6.69-8.14)	2.08(1.14-3.02)	
Resuscitation and thermal equipment	Intervention	4.79(4.01-5.57)	7.61(6.9-8.32)	7.99(7.38-8.6)	3.2(2.22-4.17)	0.52 (−0.99-2.02)
	Control	4.9(3.92-5.88)	7.98(7.38-8.58)	7.58(6.87-8.29)	2.68(1.5-3.86)	
NN cleanliness	Intervention	3.74(2.97-4.51)	6.11(5.26-6.96)	6.37(5.55-7.19)	2.63(1.51-3.75)	2.15 (0.58-3.72)
	Control	6.91(6.15-7.67)	7.75(7.03-8.47)	7.39(6.67-8.1)	0.48(−0.55-1.5)	
**MCH**						
Hygiene and emergency care	Intervention	5.69(5.17-6.21)	6.89(6.42-7.35)	7.68(7.27-8.09)	1.99(1.33-2.66)	1.25 (0.39-2.12)
	Control	7.53(7.12-7.95)	7.72(7.28-8.16)	8.27(7.9-8.65)	0.74(0.17-1.31)	
OPD drugs	Intervention	5.11(4.6-5.63)	7.26(6.65-7.87)	8.3(7.84-8.77)	3.19(2.51-3.87)	0.04 (−0.95-1.03)
	Control	5.48(4.92-6.04)	7.63(7.13-8.12)	8.63(8.17-9.09)	3.15(2.42-3.88)	
MCH oxygen availability	Intervention	2.13(1.45-2.8)	4.44(3.51-5.36)	6.26(5.43-7.08)	4.13(3.08-5.18)	2.74 (1.31-4.16)
	Control	1.63(1.07-2.19)	2.1(1.36-2.84)	3.03(2.2-3.86)	1.4(0.42-2.37)	

A mean difference of difference with confidence intervals was calculated for each of the domains by obtaining the difference between the intervention and control means for each domain at 18 months. Domains related to hygiene i.e. cleanliness (PW and NN) and hygiene and emergency care in MCH consistently showed a substantial improvement in all clinical areas. Additionally the domains nutrition and supportive care and oxygen availability in PW and MCH respectively also showed substantial improvements. These changes in the domains were bigger in the intervention than control hospitals. The domains supportive care and common drugs in NN showed a decline in the absolute mean difference although this was not substantial.

## Discussion

The purpose of this work was to develop a more nuanced picture of availability of essential hospital resources. The 11 point scale appeared intelligible to health workers in pilot testing, SAQ were typically returned completed and we exceeded our minimal sample size at each survey round indicating the feasibility of this approach. In many reports (including our own earlier report [15]) resources are simply classified as available/unavailable by observation at a single time point (equivalent to scores of 0/10 or 10/10). Using health worker reports no item was scored as either completely unavailable or fully available. Although this may in part reflect an unwillingness of respondents to give a minimum or maximum score on a likert type scale [25] it may also be a better reflection of the reality of availability over time for items such as oxygen or soap for hand washing. In the majority of domains (and for many items) at baseline availability using SAQ data was worrying with low scores (<5/10) for such basic resources. Improvement was seen for most domains during intervention, either partial (control) or full. This generalised improvement may reflect a respondent bias in a non-blinded study with repeat measures, a true secular trend or some effect of the intervention. Anecdotally the public health system did improve in a short period of strong economic growth over this period (2006 to 2007). In some domains however improvement was greater in full intervention sites. A more pronounced respondent bias, an element of regression to the mean or a true effect of the intervention might explain this. While the contribution of different effects across sites cannot be determined reliably parallel, qualitative work would suggest some effect of the intervention is plausible [18,26].

Exploratory factor analysis has proven useful in creating summary scores to assess outpatient child health services in the past [27]. Here we used, for the first time we believe, exploratory factor analysis as a data reduction technique for data collected from a self administered questionnaire assessing availability of resources in three clinical areas in hospitals. The items that loaded on each of the factors in each clinical area were found to be in general agreement with the logical groupings informing the questionnaire structure although suggesting less post-factor analysis domains than pre-specified groupings. Items associated with cleanliness in our SAQ consistently loaded onto one factor in each of the departments. Availability of oxygen and the ability to provide it at the required flow rate are items that were assumed *a priori* to be in the same domain. However in the factor analysis in PW, the two items loaded on separate factors while in NN and MCH they loaded on the same factors (factor 1 and 3 respectively). Items that were considered as first line/basic or second-line drugs in the pre-specified domains consistently loaded together in one factor in each of the clinical areas albeit with other items.

Only one item (see Additional file 1) had no factor loading > 0.3 for any single factor resulting in its elimination from aggregated analysis. Thus the factor analysis allowed the 65 remaining items across three clinical areas (PW = 34, NN = 19 and MCH = 12) in the SAQ to be aggregated into 11 domains for reporting. The interpretation of the meaning of domains is subjective and relies heavily on agreement between researchers and their understanding of the context. Further data collected with this SAQ from other sites and use of confirmatory factor analysis to assess the stability of these domains would be a more rigorous test of their value, particularly as the results of exploratory factor analysis depend on the dataset used [14].

Limitations to our study were that the health workers who responded were not randomly selected but rather those on duty during the period of the survey. In addition to this, our small number of hospitals and the fact that there were no hospitals receiving no intervention (true controls) makes it hard to generalise our findings to other settings. Secondly, as suggested above it is also possible that repeated surveys of health workers may prompt more positive responses. This is perhaps more likely in health workers who may themselves have participated in more than one survey and may feel obliged to suggest improvement (a respondent bias). Thirdly in our approach, we used an SAQ where we referred to recent availability as the ten most recent times a health worker needed a resource; there are no gold standards for periods of availability therefore, we cannot be assured of the validity of using this method although, as argued earlier, other commonly used methods typically provide an all or none result at a fixed time point. However the method does lend itself to use in surveys assessing availability of resources as an adjunct to direct observation [28].

## Conclusion

Using SAQ data to assess resource availability provides an alternative to observed point prevalence. The approach was able to demonstrate poor access to resources, change over time and variability across place. The findings suggest an intervention effect in 5 out of 11 domains. Future data collection might be collected from larger and wider samples using mobile technology (mobile SAQ), be more informative, and by providing more rapid and regular reporting help address the persistent challenges in resource availability in Kenyan hospitals.

## Competing interests

The authors declare that they have no competing interests.

## Authors’ contributions

ME was responsible for the conception of the district hospital study on which this work was based. SN, NM, CO and PA were involved in data collection and management. NM led the analysis and interpretation of data with support from PA, CO, ME, JT and EA. NM and ME prepared the initial draft manuscript. NM prepared the final manuscript after input from all authors and all authors reviewed and approved the final version of the report to be published. The funders had no role in the design, conduct, analyses or writing of this study or in the decision to submit for publication.

## Pre-publication history

The pre-publication history for this paper can be accessed here:

http://www.biomedcentral.com/1472-6963/14/282/prepub

## Supplementary Material

Additional file 1 **Factors and items which contribute to each factor in each clinical area.** This file shows detailed table of all the items in each of the post hoc domains based on the latent factors discerned from the data in each department after exploratory factor analysis. Click here for file

Additional file 2 **Means of common items per hospital at different survey time points.** This file contains a detailed table showing means of items that were common across departments at each survey. Click here for file
