# Supplementary material for: Using health worker opinions to assess changes in structural components of quality in a Cluster Randomized Trial
Source: BMC Health Serv Res. 2014 Jun 28;14:282. doi: 10.1186/1472-6963-14-282 (PMC4082497; doi:10.1186/1472-6963-14-282)
Supplement: Additional file 1 — Factors and items which contribute to each factor in each clinical area. This file shows detailed table of all the items in each of the post hoc domains based on the latent factors discerned from the data in each department after exploratory factor analysis. [file 1472-6963-14-282-S1.pdf]

Additional file 1

Table 1 Table showing factors and items which contribute to each factor in each clinical area

<table>
<tr><th>Factor</th><th>Indicators</th></tr>
<tr><td colspan="2">Paediatric Ward (PW)</td></tr>
<tr><td rowspan="3">Essential drugs/emergency resources</td><td>Consider out of ten most recent occasions the:-
*Availability of oxygen when needed</td></tr>
<tr><td>Consider out of ten most recent occasions the number of patients getting the drug from ward stock / hospital pharmacy as soon as required, without delay or paying for it before it is administered :-
*Penicillin
*Gentamicin
*IV Chloramphenicol
*Diazepam
*Quinine
*Septrin
*Amoxil</td></tr>
<tr><td>Consider out of ten most recent occasions the availability of:-
*Bolus glucose – number of times drug is immediately available to treat hypoglycaemia (within 2 minutes)
*FS Ringers / N/Saline – number of patients getting the fluids from ward stock / hospital pharmacy as soon as required, without delay and before paying for the fluid cannula or giving set.
*Other iv fluids – number of patients getting the fluids from ward stock / hospital pharmacy as soon as required, without delay and before paying for the fluid cannula or giving set.
*ORS – number of patients getting the ORS from ward stock / hospital pharmacy as soon as required on admission without delay or before paying for it.
*Blood transfusion – number of children with severe anaemia getting blood direct from blood bank before finding a donor or paying a fee.
*Blood transfusion – number of children with severe anaemia starting blood transfusion within 4 hours of prescription.</td></tr>
<tr><td>Nutrition and supportive care</td><td>Consider out of ten most recent occasions the:-
*Ability to provide oxygen at a flow of 2l/min to each individual patient.</td></tr>
</table>

	**Consider out of ten most recent occasions the adequacy of:-**<br><br>*F75 / F100/Milk feeds - number of malnourished children getting correct amount feed from ward stock / nutritionists within 4 hours of admission.<br><br>*Night feeds - number of malnourished children getting 3 hourly night feeds as recommended.<br><br>*Night feeds - number of babies being ngt fed getting 3 hourly night feeds as recommended.<br><br>*Frequent solid transition feeds - number of malnourished children getting 4 or 5 meals a day during recovery phase.<br><br>*Monitoring feed intake - number of malnourished children with an accurate feed intake chart.
Cleanliness	**Consider out of ten most recent occasions the availability of:-**<br><br>* Water from taps to wash your hands between patients<br><br>* Soap / disinfectant to clean your hands between patients<br><br>* Water from taps for mothers / caretakers to wash their hands after care-giving or eating<br><br>* Soap / disinfectant for mothers / caretakers to wash their hands after care-giving or eating
	**Consider out of ten most recent occasions:-**<br><br>* Patients bathing / washing area observed to be appropriately clean<br><br>* Patients toilets observed to be appropriately clean<br><br>* Ability to keep diarrhoea cases separately – not mixed with other patients<br><br>* Ability to isolate highly infectious cases (measles, chickenpox etc)<br><br>* Disinfectant used to clean spills of blood, stool or urine
Second-line drugs	**Consider out of ten most recent occasions the number of patients getting the drug from ward stock / hospital pharmacy as soon as required, without delay or paying for it before it is administered :-**<br><br>*Coartem<br>* Cloxacillin / Flucloxacillin<br>* Erythromicin<br>* Ceftriaxone / cefotaxime<br>* Phenobarbitone Injection
Maternity ward with newborn nursery(**NN)**	
Supportive care	**Consider out of ten most recent occasions the availability of:-**<br><br>* Phototherapy device when required for each user<br><br>* Individual cots when required to prevent sharing (except twins)

	**Consider out of ten most recent occasions the:-**<br><br>* Availability of Vitamin K – number of babies receiving routine stat dose.<br><br>* Availability of oxygen whenever needed<br><br>* Ability to provide oxygen at a flow of 1l/min to each individual patient
	**Consider out of ten most recent occasions the number of patients getting the drug from ward stock / hospital pharmacy as soon as required , without delay or paying for it before it is administered:-**<br><br>* Phenobarbitone Injection
	**Consider out of ten most recent occasions the:-**<br><br>* Number of babies with mothers who are unable to provide adequate EBM (through maternal death, maternal illness, or premature delivery) who spend 3 or more days receiving inadequate feed volumes<br><br>* Number of babies being ngt fed getting 3 hourly night feeds as recommended (night feeds).<br><br>* Number of ngt fed babies with an accurate feed intake chart(monitoring feed intake)
Common Drugs	**Consider out of ten most recent occasions the number of patients getting the drug from ward stock / hospital pharmacy as soon as required , without delay or paying for it before it is administered:-**<br><br>* Penicillin<br>* Gentamicin<br>* Cloxacillin / Flucloxacillin<br>* Ceftriaxone / cefotaxime
	**Consider out of ten most recent occasions the availability of:-**<br><br>* Bolus glucose – number of times drug is immediately available to treat hypoglycaemia (within 2 minutes)
Resuscitation and thermal equipment	**Consider out of ten most recent occasions the availability of:-**<br><br>* Warmer for resuscitation in delivery room when required<br><br>* Incubator or long term warming method when required<br><br>* Working bag and mask device when required
Cleanliness	**Consider out of ten most recent occasions the availability of:-**<br><br>* Water from taps to wash your hands between patients<br><br>* Soap / disinfectant to clean your hands between patients
Maternal and child health clinic (**MCH)**	

Hygiene and emergency care	**Consider out of ten most recent occasions the availability of:-**<br><br>* Water from taps to wash your hands between patients<br><br>* Soap / disinfectant to clean your hands between patients<br><br>* Disinfectant was used to clean spills of blood, stool or urine
	**Consider out of ten most recent occasions the number of patients getting the drug from ward stock / hospital pharmacy as soon as required , without delay or paying for it before it is administered:-**<br><br>*Diazepam
	**Consider out of ten most recent occasions the availability of:-**<br><br>* Bolus glucose – number of times drug is immediately available to treat hypoglycaemia (within 2 minutes)<br><br>* Ringers / N/Saline – number of patients getting the fluids from ward stock / hospital pharmacy as soon as required without delay and before paying for the fluid cannula or giving set.<br><br>* ORS – number of patients getting the ORS from ward stock / hospital pharmacy as soon as required without delay and before paying it.
Drugs	**Consider out of ten most recent occasions the number of patients getting the drug from ward stock / hospital pharmacy as soon as required , without delay or paying for it before it is administered:-**<br><br>* Coartem<br>* Septrin<br>*Amoxil
Oxygen availability	**Consider out of ten most recent occasions the:-**<br><br>* Availability of oxygen whenever needed<br><br>* Ability to provide oxygen at a flow of 2l/min to each individual patient

Only one item[1] in NN had no factor loading > 0.3 for any single factor resulting in its elimination from aggregated analysis.

[1] Equipment available; Consider out of ten most recent occasions the availability of:- Paediatric iv fluid drip set (with burette) when required
