# Supplementary material for: Using health worker opinions to assess changes in structural components of quality in a Cluster Randomized Trial
Source: BMC Health Serv Res. 2014 Jun 28;14:282. doi: 10.1186/1472-6963-14-282 (PMC4082497; doi:10.1186/1472-6963-14-282)
Supplement: Additional file 2 — Means of common items per hospital at different survey time points. This file contains a detailed table showing means of items that were common across departments at each survey. [file 1472-6963-14-282-S2.pdf]

Additional file 2

Means of common items per hospital at different survey time points

Item	Hospital	Baseline m(ci)	6 months m(ci)	18 months m(ci)
Water from taps to wash your hands between patients	H1	2.43(1.62-3.23)	6.83(5.86-7.81)	6.31(5.31-7.31)
	H2	6.52(5.43-7.61)	7.49(6.58-8.4)	7.88(7.15-8.61)
	H3	2.93(2.17-3.69)	2.97(2.01-3.93)	5.19(4.16-6.22)
	H4	4(3.25-4.75)	5.02(4.2-5.84)	5.15(4.27-6.03)
	H5	6.34(5.71-6.97)	5.75(4.79-6.71)	6.22(5.49-6.95)
	H6	7.9(7.34-8.45)	8.6(8.05-9.15)	8.12(7.14-9.09)
	H7	3.84(2.91-4.77)	6.1(5.23-6.96)	6.31(5.24-7.37)
	H8	8.92(8.38-9.45)	8.42(7.81-9.03)	8.24(7.66-8.82)
Soap / disinfectant to clean your hands between patients	H1	5.28(4.18-6.37)	6.52(5.47-7.57)	6.25(5.17-7.33)
	H2	6.06(4.89-7.23)	6.95(5.92-7.98)	7.68(6.84-8.52)
	H3	3.65(2.64-4.65)	3.89(2.74-5.03)	6.22(5.22-7.22)
	H4	4.74(3.77-5.71)	4.06(3.04-5.09)	6.61(5.66-7.56)
	H5	4.54(3.61-5.48)	6.68(5.6-7.76)	4.68(3.77-5.59)
	H6	6.64(5.79-7.48)	6.92(5.97-7.87)	6.44(5.28-7.61)
	H7	7.84(6.9-8.78)	8.26(7.4-9.13)	7.92(7.03-8.8)
	H8	9.22(8.66-9.79)	8.54(7.75-9.33)	8.37(7.68-9.06)
Availability of oxygen when needed	H1	3.33(2.17-4.48)	4.54(3.07-6.01)	7.79(6.94-8.63)
	H2	5.37(4.07-6.67)	9(8.42-9.57)	8.65(7.95-9.34)
	H3	5.51(4.48-6.55)	5.55(4.26-6.83)	6.61(5.3-7.91)
	H4	4.38(3.3-5.46)	7.35(6.33-8.36)	7.12(6.03-8.21)
	H5	3.57(2.41-4.74)	4.03(2.57-5.49)	3.91(2.73-5.1)
	H6	3.95(2.71-5.2)	5.87(4.61-7.12)	7.2(5.79-8.61)
	H7	3(1.91-4.09)	6.57(5.34-7.81)	4.56(3.26-5.87)
	H8	6.02(4.59-7.45)	6.27(4.94-7.6)	6.75(6.75-6.75)
Ability to provide oxygen at a flow of 2l/min(for PW and MCH) or 1l/min(NN) to each individual patient	H1	1.38(0.55-2.2)	2.95(1.62-4.28)	6.88(5.79-7.96)
	H2	4.31(3.02-5.59)	8.29(7.59-8.98)	7.53(6.6-8.46)
	H3	3.16(2.31-4)	3.25(2.12-4.38)	4.93(3.58-6.29)
	H4	3.65(2.47-4.83)	5.08(3.84-6.32)	6.2(5.07-7.34)
	H5	2.32(1.34-3.3)	2.43(1.1-3.76)	3.13(2.02-4.24)
	H6	2.31(1.15-3.48)	4.16(2.91-5.41)	5.25(3.32-7.18)
	H7	1.68(0.74-2.61)	5.16(3.82-6.5)	3.54(2.37-4.72)
	H8	1.27(0.36-2.17)	4.28(2.95-5.6)	5.1(3.95-6.25)
Bolus glucose – number of times drug is immediately available to treat hypoglycaemia (within 2 minutes)	H1	3.67(2.68-4.67)	7.61(6.53-8.68)	8.76(8.13-9.4)
	H2	7.66(6.82-8.5)	9.01(8.35-9.67)	9.23(8.68-9.79)
	H3	5.07(4.2-5.93)	7.45(6.5-8.41)	8.07(7.25-8.9)
	H4	6.83(5.91-7.76)	8.33(7.54-9.11)	8.13(7.13-9.14)
	H5	5.68(4.65-6.71)	7.41(6.39-8.43)	8.14(7.25-9.03)
	H6	7.93(6.97-8.88)	9.33(8.84-9.81)	8.57(7.67-9.47)
	H7	6.28(4.91-7.65)	8.37(7.57-9.16)	8.5(7.62-9.38)
	H8	9.28(8.62-9.95)	9.07(8.42-9.72)	8.44(7.58-9.29)
